# Mechanisms of natural resistance of Balb/c mice to experimental liver amoebiasis

**DOI:** 10.1042/BSR20182333

**Published:** 2019-05-03

**Authors:** Azucena Cortes, Mario Nequiz, Janeth Sandoval, Edith Mendoza, Marco Gudiño, Gabriel López-Velázquez, Sergio Enríquez-Flores, Emma Saavedra, Ruy Pérez-Tamayo, Alfonso Olivos-García

**Affiliations:** 1 Unidad de Investigación en Medicina Experimental, Facultad de Medicina, Universidad Nacional Autónoma de México, Ciudad de México 04510, México; 2 Grupo de Estudio en Biomoléculas y Salud Infantil, Laboratorio de EIMyT, Instituto Nacional de Pediatría, Ciudad de México 04530, México; 3 Departamento de Bioquímica, Instituto Nacional de Cardiología Ignacio Chávez, Ciudad de México 14080, México

**Keywords:** complement, Entamoeba histolytica, innate immunity, resistance to amoebiasis

## Abstract

*Entamoeba histolytica* is the parasite responsible for human amoebiasis. The analysis of the natural resistance mechanisms of some rodents to amoebic liver abscess (ALA) may reveal alternative pathogenicity mechanisms to those previously discovered in the experimental model of ALA in hamsters. In this work the natural resistance of BALB/c mice to ALA was explored by performing: (i) *in vivo* chemotaxis analysis with a specifically designed chamber; (ii) *in vitro* amoebic survival in fresh and decomplemented serum; (iii) histological temporal course analysis of ALA development in mice with different treatments (hypocomplementemic, hyperimmune and treated with iNOS and NADPH oxidase inhibitors) and (iv) mouse liver amoebic infection by both *in situ* implantation of ALA from hamsters and inoculation of parasites into the peritoneal cavity. The results show that *E. histolytica* clearance from the mouse liver is related to a low chemotactic activity of complement, which results in poor inflammatory response and parasite inability to cause tissue damage. Also, the absence of amoebic tropism for the mouse liver is correlated with resistance to experimental liver amoebiasis.

## Introduction

*Entamoeba histolytica* is the parasite responsible for human amoebiasis. This illness causes near 100000 annual deaths worldwide and is prevalent in poor countries [1]. Notwithstanding that metronidazole diminished mortality caused by amoebiasis, there are some reports of parasite resistance to this drug and toxicity on microbiota, which lead to dysbiosis [2,3]. For these reasons it is very important to expand the understanding of the pathogenicity mechanisms of the infection. Such new information may also help in the development of new therapeutic alternatives.

Experimental amoebic liver abscess (EALA) in hamsters has allowed the identification of some pathogenic mechanisms, which led to the proposal of several potential amoebic targets. In contrast, different rat and mouse strains are resistant to EALA development [4]. Innate immunity may play an important role in such resistance since in most of them the parasite is cleared before the specific immune response. Recently, it was determined that serum complement of Wistar rats is lethal for the parasite and is involved in their resistance to EALA [5]. Furthermore, Balb/c mice have been used to study the EALA development; however, contrasting results have been published showing early as well as late clearance of the parasites (from 1 to 7 days) [6–10]. The discrepancy in clearance time of *E. histolytica* from the liver of Balb/c mice may be due to the use of different parasite inocula (0.75 × 10^6^ to 5 × 10^6^) and/or mechanical damage caused by the needle during the intrahepatic injection of parasites.

In this work, the resistance mechanism of Balb/c mice to EALA was explored by using the intraportal parasite administration route which does not cause mechanical damage and by injecting 1 × 10^6^ parasites/100 g body weight, an amount that is comparable with that used for EALA in hamsters [11]. Our results suggest that resistance of Balb/c mice to EALA is due to: (i) a low chemotactic activity of complement, which results in a poor inflammatory response and concomitant parasite inability to cause tissue damage and (ii) invasion impairment due to the absence of tropism for mouse liver.

## Materials and methods

### Ethics statement

All experiments involving animals were performed in strict accordance with the Mexican Law for the Production, Care and Use of Laboratory Animals (NOM-062-ZOO-1999). All animal procedures were carried out under the protocol number 091–2016, approved by the Institutional Animal Care and Use Committees of the Facultad de Medicina, Universidad Nacional Autónoma de México. All efforts were made to minimize animal suffering.

### Parasites and virulence

Axenic cultures of virulent *E. histolytica* strain HM1-IMSS were maintained in TYI-S-33 medium according to standard protocols. Virulence was defined as the ability of 1 × 10^6^ trophozoites to produce multiple liver abscesses in 4/4 hamsters (*Mesocricetus auratus*, 100 g weight) 7 days after intraportal inoculation. Such virulence was maintained by passing axenic trophozoites through hamster livers once a month, recovering the parasites from 7-day-old abscesses and again growing them axenically.

### Cobra venom factor purification

Cobra venom factor (CVF) was purified from crude commercial preparations of *Naja haje* venom (Sigma, St. Louis, MO, U.S.A.) by size exclusion and ion-exchange chromatography [12]. Hypocomplementemic activity of purified CVF was evaluated *in vivo* by intraperitoneal injection of 100 μg CVF in a rat according to Van den Berg et al. [13]. After 1, 2, 3, and 4 days of CVF injection, blood (1.5 ml) from the tail vein of the rat was obtained and the *in vitro* amoebic lytic effect of the sera was determined incubating 1 × 10^6^ trophozoites/ml of each serum for 2 h at 36.5°C. After this period, cell viability was determined by Trypan Blue exclusion.

### Acute amoebic liver infection in mice

Either male or female Balb/c mice (25 g) from our in-house colony were anesthetized with ketamine (80 mg/kg body weight)–xylazine (5 mg/kg body weight), the abdominal cavity was entered and 0.25 × 10^6^ axenic trophozoites suspended in 50 µl of phosphate-buffer saline (PBS) were intraportally injected. Each experiment was performed in four animals. At different times after the intraportal injection, the animals were anesthetized with ether, killed by cardiac bleeding and the livers were removed, weighed, sliced for gross inspection, fixed in 3.7% formaldehyde in PBS and all liver lobes processed for histological study and stained with Periodic Acid–Schiff (PAS) stain. For comparison, parallel groups of hamsters were infected and treated likewise. The level of inflammatory response in mice and hamsters was determined at 3 and 12 h post-infection by counting leukocytes present in 20 well-delimitated inflammatory foci. A 12-h post-infection maximum time was selected; beyond that, it is not possible to distinguish in the hamster liver well-preserved leukocytes because most of them have been lysed.

### Acute amoebic liver infection in hypocomplementemic mice

CVF (15 µg) was injected intraperitoneally in a group of mice, and 24 h later, 0.25 × 10^6^ trophozoites were injected into their portal vein. The animals were killed at 6, 24, and 72 h after parasite injection and their livers processed for histologic analysis, as described above.

### *In vivo* chemotaxis of leukocytes

Virulent trophozoites of *E. histolytica* (5 × 10^4^) were resuspended in 60 µl of fresh or heat-decomplemented hamster or mouse serum and placed in specially designed small glass chambers (0.3 × 1.6 cm). The chamber was covered with a polycarbonate membrane with 3-µm pores (Neuro Probe, Inc.) and sealed with an elastic O-ring. Then the chambers were introduced into the peritoneal cavity of different mice groups. After 6 h, the leukocytes that entered the chambers were resuspended with Türk solution and counted in a Neubauer chamber. All this procedure was performed under aseptic conditions and was repeated four times.

### *In vitro* amoebic growth in sera

Virulent trophozoites of *E. histolytica* (1.25 × 10^5^) were resuspended in 600 µl of mice serum previously decomplemented by heat (56 °C for 30 min) and sterilized by filtration (0.22 µm membrane pore). The samples were incubated at 36.5 °C in sterile glass vials (12 × 32 mm) and after 24, 48 and 72 h amoebic growth and viability was determined by Trypan Blue exclusion. Amoebic viability was compared with the control (TYI-S-33 medium). This procedure was repeated three times.

### Amoebic lytic effect of sera

Virulent amoebic trophozoites (1 × 10^6^) obtained from cell cultures were incubated for 2 h at 36.5 °C in a rocker with 1 ml of fresh serum from hamster, rat, mouse and human. After this period, cell viability was determined by Trypan Blue exclusion. The amoebic viability of samples was compared with the control (TYI-S-33 medium).

### Acute amoebic liver infection in hyperimmune mice

Either male or female Balb/c mice (25 g) were injected subcutaneously and intramuscularly with amoebic lysates (0.5 × 10^6^/100 µl PBS) plus 50 µl complete Freund’s adjuvant. After 1 week, the same animals were immunized using a similar protocol with amoebic lysates *plus* incomplete Freund’s adjuvant and in the third week, they were immunized only with amoebic lysates. After 5 days the presence of antiamoebic antibodies was evaluated, three animals were bled and their sera used for Western blot analysis.

On the other hand, a week after the last immunization, 0.25 × 10^6^ amoebae were injected by intraportal route as described above and after 6, 24 and 72 h, three animals for each time were killed and their livers processed for histology as mentioned above. The level of inflammatory response at each time was determined by counting the leukocytes present in 20 well-delimitated inflammatory foci.

### Acute amoebic liver infection in hyperimmune mice treated with iNOS, NADPH oxidase and complement inhibitors

Three different groups of mice previously immunized with amoebic lysates were intraperitoneally treated 8 h before the parasite injection with: (i) vehicle (25 µl of ethanol 2 %), (ii) 0.41 mg of apocynin (inhibitor of NADPH-oxidase) and (iii) 0.10 mg of mercapto-ethylguanidine (iNOS inhibitor). In addition, other group received only a CVF dose (15 µg) 24 h before amoebic infection. Then 0.25 × 10^6^ trophozoites were injected by intraportal route as mentioned above and treatment with inhibitors were continued every 8 h until animal killing. Three animals for each time were killed after 6, 24 and 72 h of parasite injection and their livers processed for histology as mentioned above. The level of inflammatory response at each time was determined by counting the leukocytes present in 20 inflammatory foci.

### Acute amoebic liver infection in mice infected by the intraperitoneal route

A group of mice was injected intraperitoneally with 3 × 10^6^ trophozoites suspended in 1 ml of PBS. The animals were killed by anesthesia overdose at 6, 12, 24 and 48 h after parasite injection and their livers removed and processed for histologic analysis, as described above.

### Mouse liver amoebic infection by implantation of amoebic liver abscess of hamsters

The smallest hamster liver lobule with 24-h amoebic liver abscess (ALA) was dissected as described above and implanted under the liver of a live normal mice and hamsters. After 24, 48 and 72 h both, the proper and implanted livers were processed for histologic analysis, as described above. The procedure was performed in three animals/group/time.

### Statistical analysis

Where indicated, statistical test used is mentioned in the figure legend and was performed using GraphPad Prism version 5.00 for Windows (GraphPad Software; San Diego, CA, U.S.A.).

## Results

### *E. histolytica* is cleared from mouse liver before 72 h

**Figure 1:**
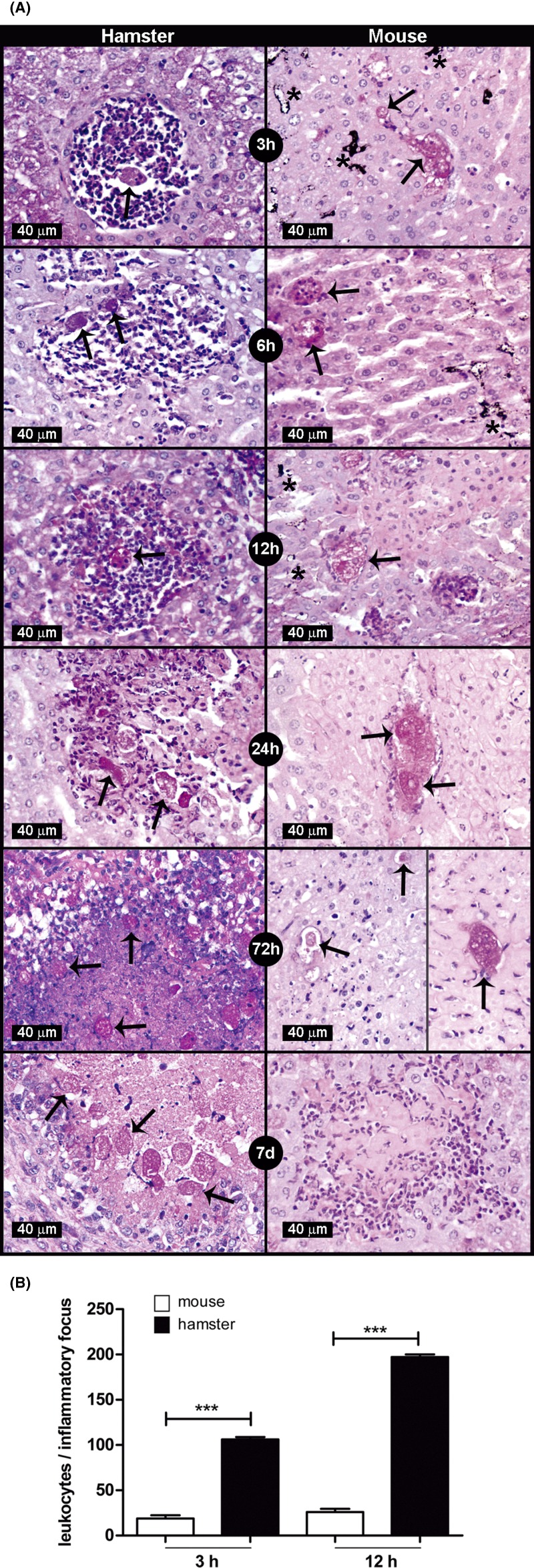
*E. histolytica* survival in hamster and mouse livers (**A**) Amoebae (1 × 10^6^/100 g body weight) were injected into the portal vein of normal hamsters and mice (four/group) and after 3, 6, 12, 24, 72 h and 7 days the livers were removed, fixed and tissue slices were stained with PAS. In hamster liver from 3 to 12 h, amoebae were surrounded by abundant PMNs. From 24 h up to 7 days, many well-preserved amoebae with intense inflammatory infiltrate and progressive lytic necrosis were observed. In contrast, in mouse liver, from 3 up to 72 h well-preserved amoebae were surrounded by scarce or no inflammatory infiltrate, with coagulative necrosis; the glycogen exhaustion in amoebae after 72 h was striking. The ischemia was present as early as 3 h demonstrated by the lack of perfusion of India Ink (*), whereas after 7 days only residual lesions without amoebae were observed. Amoebae are indicated with arrows. (**B**) At 3 and 12 h after parasite inoculation, the inflammatory level was quantitated by determining the number of leukocytes in the amoebic surrounding. The inflammatory response increased considerably only in the hamster liver. Statistical analysis was done by using one-way ANOVA, the comparison between the groups was done according Bonferroni’s correction. Asterisks indicate significant differences between groups as indicated; ***P≤0.001. Data are presented as mean ± S.E.M.

To determine the parasite clearance time from mouse and hamster livers, a time course of amoebic liver infection was analyzed. In mice livers, well-preserved parasites and their debris were visible in ischemic areas from 3 up to 72 h, with minimal or no inflammatory cells. In particular, low PAS staining of both debris and ghost amoebae were typical at the 72 h time. Ischemia was present from 3 h to 7 days as demonstrated by lack of India Ink perfused. Also, after 7 days, rare residual lesions were observed (Figure 1A). These results are in contrast with the findings in hamster liver which show well-preserved amoebae surrounded by abundant inflammatory infiltrate with progressive parasite proliferation and necrosis (Figure 1A). In addition, the number of inflammatory cells during early amoebic liver infection (3 and 12 h) determined with a computer software, showed a significant increment in inflammatory response in hamsters in comparison with mice (Figure 1B).

### Complement is not involved in the amoebic clearance from mice liver

**Figure 2:**
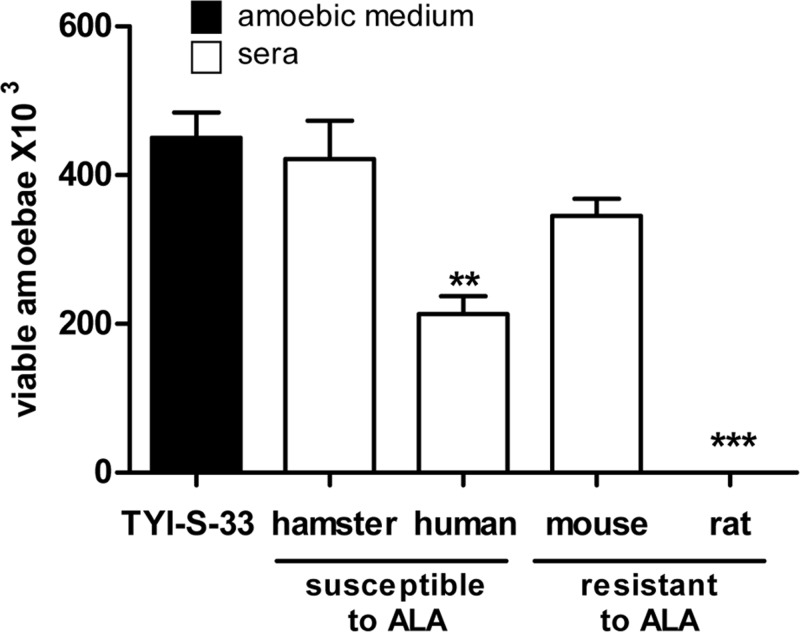
*E. histolytica* susceptibility to hamster, mouse, human and rat fresh sera Amoebae (1 × 10^6^/ml) were incubated with hamster, mouse, human and rat fresh sera (three independent sera/species) either male or female, and after 2 h the viability was determined. The amoebicidal activity of mouse serum is similar to that of susceptible species to ALA. Statistical analysis was done by using one-way ANOVA with Dunnet post-test. Asterisks indicate significant differences respect to the control TYI-S-33 medium as indicated; **P≤0.01, ***P≤0.001. Data are presented as mean ± S.E.M.

To explore if complement is involved in the mice natural resistance to amoebic liver infection, parasites were injected into the portal vein of mice decomplemented with CVF. Although hypocomplementemia allowed 100% *in vitro* amoebic survival in mouse serum, it did not prolong amoebic survival in mice livers (data not shown). In addition, the amoebicidal activity of normal fresh mice sera did not correlate with resistance to EALA, since their levels were very similar to that of human and hamster which are susceptible to EALA but quite different from rat, that is resistant to EALA, and showed 100% complement amoebicidal activity (Figure 2).

### Leukocyte chemotaxis of fresh hamster serum is higher than that of mouse and is due to complement

**Figure 3:**
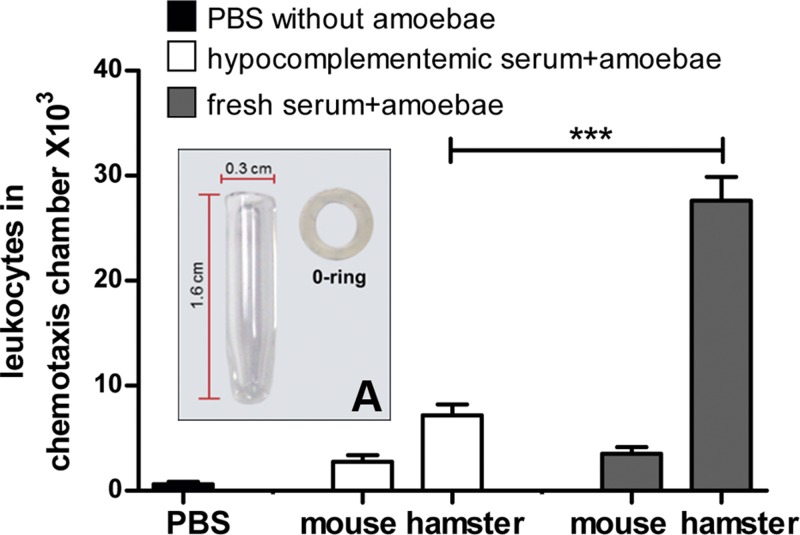
Chemotaxis of mice peritoneal leukocytes by hamster and mouse fresh sera *plus* amoebae Amoebae (5 × 10^4^/60 µl) mixed with hamster or mouse fresh sera either male or female (four animals/species) were introduced into the mice peritoneal cavity inside a chemotaxis glass chamber (**A**) and incubated for 6 h; afterwards, the chamber was removed and leukocytes inside it were counted. Only fresh hamster *plus* amoebae showed the highest chemotactic activity. Statistical analysis was done by using one-way ANOVA, the comparison between the groups was done according Bonferroni’s correction. Asterisks indicate significant differences between groups as indicated; ***P≤0.001. Data are presented as mean ± S.E.M.

To explore if complement is involved in the lower leukocyte chemotaxis in mouse livers, amoebae were resuspended in hamster or mice sera, placed into an on-purpose designed small glass chamber (inset in Figure 3) and introduced into the mouse peritoneal cavity. After 6 h of incubation, the leukocyte chemotaxis level (quantitated as the number of leukocytes inside the glass chamber) was similar in amoebae *plus* hamster or mouse heat-decomplemented sera. In contrast, the chemotaxis level of amoebae *plus* hamster fresh sera was approximately seven times higher than amoebae plus mouse fresh sera (Figure 3). This result suggests that amoebae are able to activate the hamster (but not the mouse) complement system, through the alternate pathway as occurs with human complement [14].

### The inflammatory infiltrate was stimulated by pre-immunization with amoebic lysates in mice

**Figure 4:**
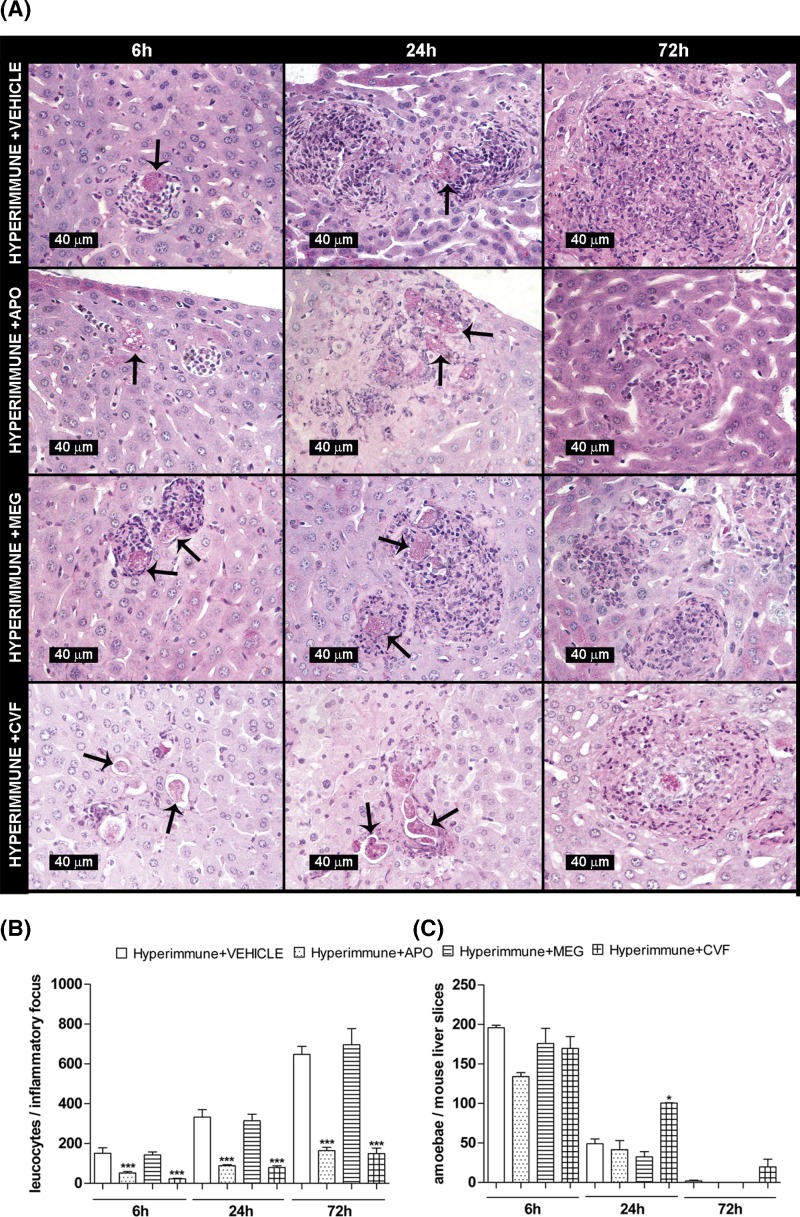
Amoebic survival in mouse liver with high inflammatory infiltrate induced by pre-immunization (**A**) Amoebae (0.25 × 10^6^) were injected into the portal vein of different groups of hyperimmune mice (four/group) treated with: 1) vehicle, 2) apocynin (APO), 3) mercapto-ethylguanidine (MEG) and 4) CVF. After 6, 24 and 72 h of infection, the livers were removed, fixed, and tissue slices were stained with PAS. As in normal mouse, in all groups the parasite was cleared before 72 h. (**B**) Leukocytes surrounding amoebae were counted using a computer software. After 6, 24 and 72 h of infection, the highest leukocyte amount was observed only in vehicle and MEG groups. (**C**) Well-preserved amoebae present in all liver slices of all groups were manually counted. The number of parasites was gradually reduced and it was similar in all groups. Statistical analysis was done by using one-way ANOVA; the comparison between groups was done according Bonferroni’s correction. Asterisks indicate significant differences respect to the hyperimmune mice as indicated; *P≤0.05, ***P≤0.001. Data are presented as mean ± S.E.M.

In order to stimulate leukocyte chemotaxis during amoebic liver infection in mice, the classical complement pathway was stimulated by antiamoebic antibodies generated by repeated immunizations with amoebic lysates. In the livers of immunized mice infected with amoebae, the presence of antiamoebic antibodies (verified by Western blot), stimulated chemotaxis of the inflammatory cells to the parasite (Figure 4A,B).

### Complement, nitric oxide and reactive oxygen species were not involved in the amoebic clearance in the liver of hyperimmune mice

The livers of hyperimmune mice were characterized by abundant inflammatory infiltrate surrounding the parasite from 6 up to 72 h (Figure 4B). However, despite this reaction, amoeba still disappear before 72 h (Figure 4A,C).

To determine if ROS or NO^•^ derived from inflammatory cells were responsible for the amoebic clearance in the livers of immunized mice, amoebae were injected in animals treated with apocynin (NADPH oxidase inhibitor) or mercapto-ethylguanidine (iNOS inhibitor). Neither ROS nor NO^•^ are involved in the amoebic clearance from the liver of immunized mice since they did not prolong the parasite survival after 72 h (Figure 4A,C). The apocynin efficacy was evident because inflammatory infiltrate, which is stimulated by ROS, was considerably diminished in this group (Figure 4B). However, under these conditions, complement is another factor that could be responsible for the early amoebae elimination.

To explore the latter possibility, amoebic survival time in the liver of hyperimmune-hypocomplementemic mice were determined by using CVF. Under these conditions, complement is not involved in the parasite clearance since hypocomplementemia did not prolong amoebic survival after 72 h (Figure 4A,C). Also, the lack of inflammatory infiltrate confirms that the absence of chemotactic molecules derived from complement is responsible for the absence of inflammatory response of normal Balb/c mice during amoebic liver infection (Figure 4B).

### Mouse serum supports *in vitro* amoebic survival for 48 h

**Figure 5:**
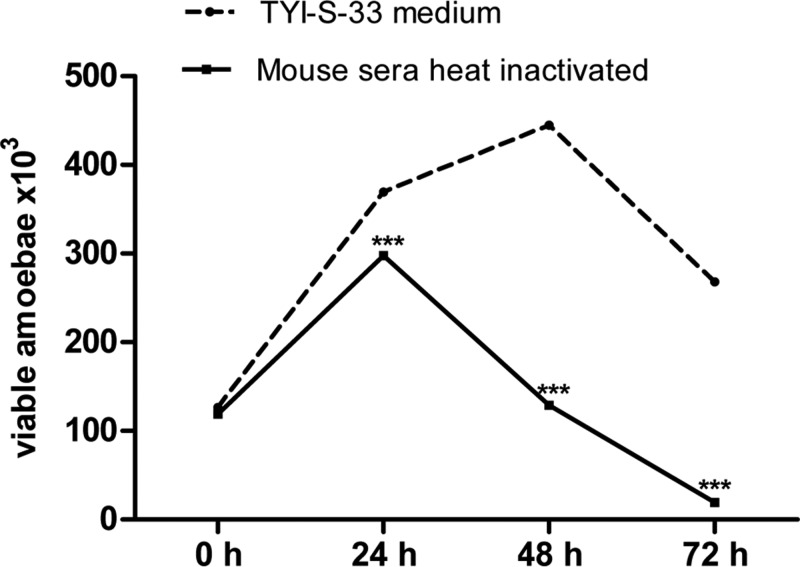
*In vitro* amoebic survival in inactivated mouse serum *E. histolytica* (1.25 × 10^5^/600 µl) trophozoites were incubated in TYI-S-33 medium or heat-decomplemented mice sera at 36.5°C, and after 24, 48 and 72 h the viability was determined. Within 24 h in mice sera, amoebae replicate but viability was gradually lost. Statistical analysis was done by using one-way ANOVA, the comparison between the groups was done according to Bonferroni’s correction. Asterisks indicate significant differences respect to TYI-S-33 medium as indicated; ***P≤0.001. Data are presented as mean ± S.E.M.

To determine if sinusoidal serum may support parasite survival, the *in vitro* amoebic growth in mouse serum inactivated by heat was evaluated. At 24 h of incubation, the mouse serum allowed amoebae replication; afterwards there was a gradual loss of viability that contrasted with amoebic growth in culture medium (Figure 5).

### *E. histolytica* injected into the mouse peritoneum remains below the liver capsule and disappears after 48 h

**Figure 6:**
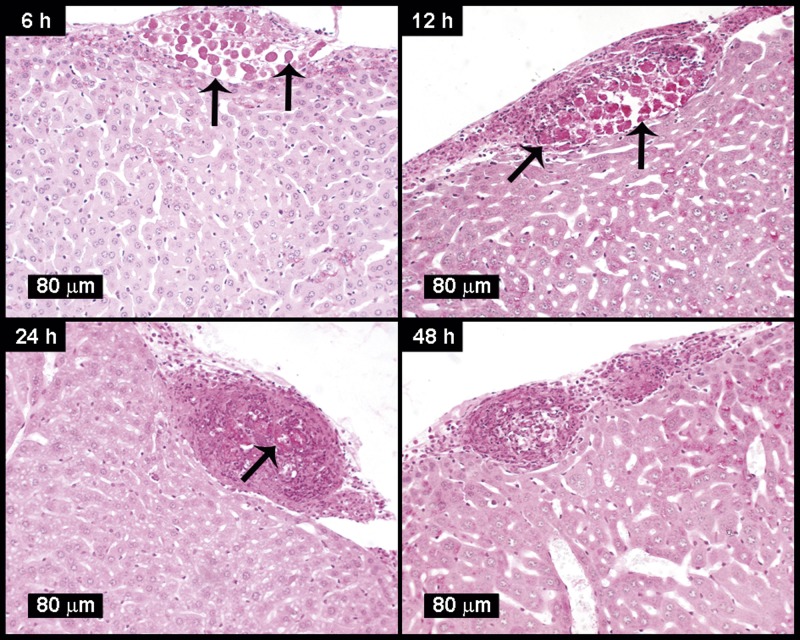
*E. histolytica* survival in mouse liver infected through intraperitoneal route Amoebae (3 × 10^6^/1 ml PBS) were injected into the peritoneal cavity of normal mice (four/group) and after 6, 12, 24 and 48 h their livers were removed, processed for histology and stained with PAS. At 6 and 12 h the parasite was able to penetrate the Glisson capsule with scarce inflammatory response. At 24 h the parasites do not invade the liver parenchyma and are encapsulated by granulation tissue and cleared after 48 h in the absence of apparent liver ischemia. Arrows indicate well-preserved parasites.

In order to test amoebic survival in mouse liver in the absence of ischemia, the parasite was injected into the mouse peritoneal cavity (near the liver location) to allow amoebic invasion through the liver capsule. After 6 h of innoculation, the parasites were observed in the liver surface below the capsule without leukocytes presence. After 12 h, trophozoites look well preserved surrounded by few inflammatory cells and without necrosis. At 24 h, vacuolated and fragmented amoebae were encapsulated by granulation tissue whereas at 48 h only residual lesions without parasites were observed (Figure 6).

### Adapted amoebae show tropism for hamster liver but not for mouse

**Figure 7:**
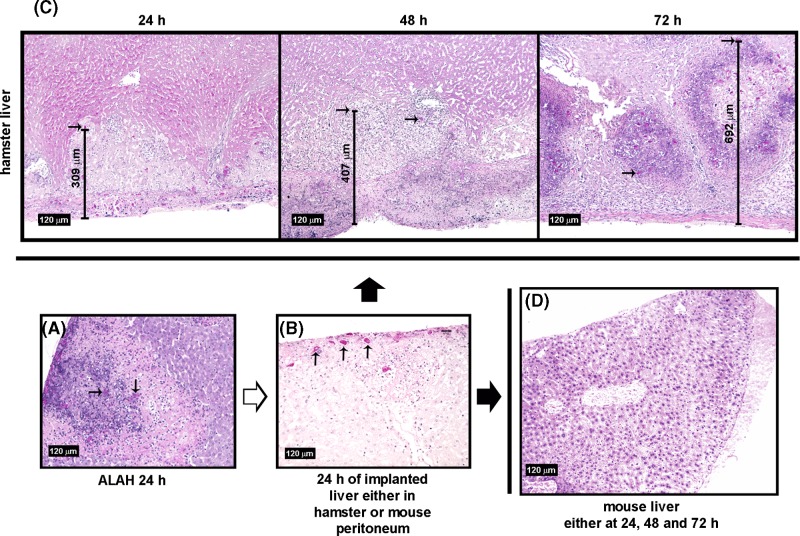
Hamster and mouse liver infection by peritoneal implantation of an infected hamster liver lobule Amoebae (1 × 10^6^/100 g body weight) were injected into the portal vein of normal hamsters and after 24 h the smallest lobule (**A**) was implanted under the liver of naive mice and hamsters. After 24, 48 and 72 h, both the implanted and host livers were processed for histology and PAS staining. In both animals and after 24 h, the amoebae from the implanted lobule moved to the periphery (**B**). In the host hamster liver, amoebae were able to reach the liver parenchyma (∼10.3 µm/h), spread fast and cause extensive tissue damage (**C**). In contrast, despite amoebae reached the edge of the implanted lobule in mice (**B**), they neither penetrated the Glisson capsule nor invade the liver parenchyma (**D**). Arrows indicate well-preserved parasites.

To determine if amoebae adapted to ALA could penetrate the livers of non-infected mouse or hamster, trophozoites were injected into the portal vein of a group of hamsters. After 24 h, the animals were killed and the small lobe liver was excised and implanted below the liver of live and naive mice and hamster animals. In the lobe of ALA in hamsters after 24 h, amoebae were confined to the inflammatory foci (Figure 7A) and when the liver was implanted in both types of animals for 24 h, some trophozoites moved to the lobule periphery (Figure 7B). Additionally, in the live naive hamster amoebae penetrated the liver parenchyma up to 309 µm in 24 h and after 3 days they spread fast (692 µm), causing larger lesions than when they were directly injected into the peritoneum (Figure 7C). Remarkably, in the live naive mouse, the amoebae located at the edge of the implanted hamster liver were unable to penetrate the mouse liver parenchyma (Figure 7D).

## Discussion

Several mouse strains have been used to analyze only the early steps of EALA because they did not develop progressive lesions. However, differences in amoebic clearance times have been observed among them [4]. Such variability in the results may be influenced by: (a) the immune status of the animal, (b) the mechanical damage caused by the needle during direct inoculation to the liver and (c) different amounts of parasite inoculum. For example, *in vitro* amoebic toxicities of complement and oxygen, that are present during early EALA, varies depending on the density of parasites (personal observation). In this work, such discrepancies were solved by: (i) using the intraportal parasite administration route that does not cause direct mechanical damage in the liver, and (ii) injecting 1 × 10^6^ parasites/100 g body weight, a parasite load comparable with that used in the EALA in hamsters [11]. Under these conditions, parasites disappeared from the livers of Balb/c mice after 72 h of infection (Figure 1A).

The potent amoebicidal activity of complement in Wistar rats is responsible for parasite elimination before 6 h [5]. In contrast, the amoebicidal activity of complement in Balb/c mouse is not involved in the parasite clearance from liver since hypocomplementemia did not modify the survival time of the parasite (72 h). Also, the *in vitro* amoebicidal activity of mouse complement was similar to that of hamster and human which are susceptible to liver amoebiasis (Figure 2).

In the hamster liver, ischemia generated by the abundant inflammatory response favors parasite survival by diminishing the affluence of blood dangerous molecules like oxygen, complement and antibodies [15–17]. Likewise, in the livers of Balb/c mice, ischemia was present as early as 3 h as demonstrated by the non-perfusion of India Ink in the parasite surroundings; such phenomenon despite that amoebae did not stimulate an inflammatory response (Figure 1A). Furthermore, it seems that ischemia may promote amoebic survival up to 72 h, since when it was absent when parasite invasion was due through the liver capsule (intraperitoneal amoebic inoculation), the amoebic survival time diminished to 24 h (Figure 6).

On the other hand, it has been demonstrated in the EALA in hamsters that the presence of abundant leukocytes is a requirement for amoebic survival [17]. In contrast, amoebic infection in Balb/c liver was characterized by the absence of an inflammatory infiltrate (Figure 1A,B). Pre-immunization of mice with total amoebic antigens allowed increase in the inflammatory infiltrate in the parasite surroundings, by activation of the classical pathway of complement since hypocomplementemia inhibited this effect (Figure 4A,B). Also, in the Balb/c peritoneum, hamster complement (but not the Balb/c one) *plus* amoebae attracted abundant leukocytes (Figure 3). Such results support the hypothesis that the absence of peptides derived from Balb/c complement is responsible for the lack of inflammatory response during EALA in mice.

Conversely, *in vitro* experiments have shown that oxidants derived from inflammatory cells like NO^•^, H_2_O_2_ and HOCl have potent amoebicidal activities [18,19]. Also, it has been observed that abundant inflammatory infiltrate during EALA in several mice strains and it has been proposed that oxidants produced by these cells may be responsible for the amoebic clearance [9,20–22]. In the EALA of pre-immunized Balb/c mice which has increased inflammatory infiltrate, such molecules do not participate in the amoebic clearance since specific inhibitors for NADPH oxidase (apocynin) and nitric oxide synthase (mercaptoethylguanidine), which produce ROS and NO^•^ respectively, did not prolong amoebic survival (Figure 4A,C). Although no myeloperoxidase inhibitor was used in the present study, it is thought that HOCl production was not present in animals treated with apocynin because H_2_O_2_ is required for its synthesis. In addition, it has been reported that in EALA in Balb/c mice, trophozoites remain until the fourth day, despite the increase in inflammation induced by an analog of prostaglandin E2 (PGE2) [10] as well as iNOS protein and NO^•^ (determined as nitrite blood levels) during the first 12 h of infection [9]. The lack of effect of the PGE2 analog to promote liver abscess in Balb/c mice is striking since it induces amoebic survival, inflammation and tissue damage in both, implanted human intestine in mouse and hamster liver, which are susceptible to amoebiasis [23,24].

Furthermore, in Balb/c and SCID mice, it has been suggested that neutrophils play a protective role from amoebic liver infection, since liver amoebic abscess formation is higher in neutropenic animals [6,25]. However, since parasites in the lesions were not counted and they disappear in later stages of infection (in both normal or neutropenic animals), the reparation of hepatic damaged areas rather than a direct amoebicidal activity may be a plausible explanation for such protective function. In this work the lack of lytic necrosis in ischemic hepatocytes contacting amoebae up to 72 h was remarkable (Figure 1A). This phenomenon could be explained by the presence of sinusoidal α-2-macroglobulin, which diminishes *in vitro* cytolysis of hepatocytes by amoebae because of its inhibitory activity on amoebic cysteine proteinases [26]. This is in contrast with the lytic necrosis observed in Balb/c liver by Velazquez et al. [6], that may be due to a higher proteolytic activity resulting from abundant locally injected parasites (5 × 10^6^) that exceed the inhibitory capacity of α-2-macroglobulin.

On the other hand, it is known that a reductive environment is an absolute requirement for amoebic proliferation [27] and reductive activity is held only by live cells. In human liver abscess, parasites remain at the edge of the lesions but not in the necrotic central zone which lacks host live cells [28]. Another reason for the parasite to proliferate and stay at the boundaries of the lesions could be for glucose availability from the host, since glycolysis is the only metabolic pathway of the parasite to produce carbon skeletons for anabolism, reductive intermediaries, ATP and other molecules [29]. In this regard, in the EALA in Balb/c mice after 72 h inoculation, glycogen was diminished in well-preserved parasites and their debris, suggesting intracellular glucose exhaustion before amoebic death. Glycogen decreased content has been also observed *in vitro* when *E. histolytica* was cultured in glucose-deprived medium [29]. Certainly, glucose exhaustion cannot be the only reason for amoebic death, since addition of glucose to Balb/c serum under *in vitro* conditions did not prolong amoebic survival beyond 72 h. In summary, the lack of a reductive environment in ischemic areas of EALA in Balb/c mice and the ability of sinusoidal plasma to support amoebic survival only for 72 h (Figure 5), which may include glucose exhaustion, may explain, at least in part, amoebic survival up to 72 h.

On the other side, amoebic motility is an important function associated with pathogenicity [30]. Many molecules derived from inflammatory cells and extracellular matrix like TNF-α [31], IL-8 [32], C5a [33] and fibronectin fragments [34] have chemotactic activity on parasites and it has been considered that they play an important role in intestine invasion and the spread of infection to other organs. In the present work, amoebae present in a lobe of 24 h EALA of hamster (adapted amoebae) implanted into the peritoneal cavity of a live naive hamster, moved to the lobule periphery, spread fast into the host liver parenchyma (∼10.3 µm/h) and caused extensive tissue damage (Figure 7). Despite the fact that the amoebic chemotactic displacement is approximately ten times less than that observed *in vitro* on agar using fresh amoebic medium [35], the inflammatory infiltrate and the extracellular matrix during hamster liver infection may be obstacles for free amoebic movement. In contrast, amoebae present in a similar lobule mounted on the liver of live Balb/c mouse, can enter its capsule but were unable to penetrate the liver parenchyma (Figure 7). Such discrepancies may be due to unknown chemotactic components to target the parasite delivered by the liver and/or inflammatory cells from hamster but absent from Balb/c mouse. Whatever is the case, this result suggests that *E. histolytica* shows varying tropism to the liver of resistant and susceptible animals to amoebic liver infection, as has been proposed to *E. histolytica* subpopulations for different human organs [36].

Taken together, resistance of Balb/c mouse to experimental amoebic liver infection is due to the lack of complement activation that leads to a poor inflammatory response. Under these conditions, ischemia generated by sinusoidal blood occlusion by parasites and detained plasma allow parasite survival for only 72 h. Also, the absence of amoebic tropism for the liver of Balb/c mice avoids amoebic spreading, which may also contribute to the natural resistance of this rodent to experimental liver amoebiasis.

## Abbreviations

ALA: amoebic liver abscess
CVF: cobra venom factor
EALA: experimental ALA
iNOS: inducible nitric oxide synthase
PAS: Periodic Acid–Schiff
PBS: phosphate-buffer saline
PGE2: prostaglandin E2
TNF-α: tumos necrosis factor alpha
